# Validation of a UPLC-MS/MS Method for Multi-Matrix Biomonitoring of *Alternaria* Toxins in Humans

**DOI:** 10.3390/toxins16070296

**Published:** 2024-06-28

**Authors:** Lia Visintin, María García Nicolás, Sarah De Saeger, Marthe De Boevre

**Affiliations:** 1 Centre of Excellence in Mycotoxicology and Public Health, Faculty of Pharmaceutical Sciences, Ghent University, B-9000 Ghent, Belgium; 2 Department of Analytical Chemistry, Faculty of Chemistry, Regional Campus of International Excellence “Campus Mare Nostrum”, University of Murcia, E-30100 Murcia, Spain; 3 Department of Biotechnology and Food Technology, Faculty of Science, Doornfontein Campus, University of Johannesburg, Gauteng 2028, South Africa

**Keywords:** *Alternaria* toxins, multi-matrix, UPLC-MS/MS, human biomonitoring, VAMS

## Abstract

Mycotoxins, natural toxins produced by fungi, contaminate nearly 80% of global food crops. *Alternaria* mycotoxins, including alternariol (AOH), alternariol monomethylether (AME), and tenuazonic acid (TeA), present a health concern due to their prevalence in various plants and fruits. Exposure to these toxins exceeds the threshold of toxicological concern in some European populations, especially infants and toddlers. Despite this, regulatory standards for *Alternaria* toxins remain absent. The lack of toxicokinetic parameters, reference levels, and sensitive detection methods complicates risk assessment and highlights the necessity for advanced biomonitoring (HBM) techniques. This study addresses these challenges by developing and validating ultra-high performance liquid chromatography method coupled with tandem mass spectrometry to quantify AOH, AME, TeA, and their conjugates in multiple biological matrices. The validated method demonstrates robust linearity, precision, recovery (94–111%), and sensitivity across urine (LOD < 0.053 ng/mL), capillary blood (LOD < 0.029 ng/mL), and feces (LOD < 0.424 ng/g), with significantly lower LOD for TeA compared to existing methodologies. The application of minimally invasive microsampling techniques for the blood collection enhances the potential for large-scale HBM studies. These advancements represent a step toward comprehensive HBM and exposure risk assessments for *Alternaria* toxins, facilitating the generation of data for regulatory authorities.

## 1. Introduction

Mycotoxins, naturally occurring toxic compounds produced by certain fungi, present a challenge to global food safety and public health. Among these, toxins produced by *Aspergillus*, *Fusarium*, *Alternaria*, and *Penicillium* are particularly concerning due to their prevalence in a wide array of food crops. Notably, Eskola et al. (2020) [1] highlighted that nearly 80% of global food crops are tainted with some level of mycotoxin contamination. This situation is compounded by climate change, which is expected to increase the spread of mycotoxins in Europe, thus escalating the health risks associated with their exposure [2]. Adding to the concern, the most recent annual report from the European Rapid Alert System for Food and Feed (RASFF) underscores mycotoxins as a significant hazard category, highlighting the issue as a major cause of economic loss and emphasizing the necessity for vigilant monitoring and regulatory action to safeguard public health [3].

*Alternaria alternata*, a species of particular concern, secretes mycotoxins such as alternariol (AOH), alternariol monomethylether (AME), and tenuazonic acid (TeA), predominantly affecting numerous crops, fruits, and vegetables. The European Food Safety Authority (EFSA) reports indicate that dietary exposure to TeA significantly exceeds that of other *Alternaria* toxins within the European population. Alarmingly, exposure levels for infants and toddlers surpass the established threshold of toxicological concern (TTC), indicating a potential health threat. For the adult population, both high dietary exposure scenarios (upper bound and 95th percentile) for AME and AOH are above the TTC, underscoring the public health risk posed by these contaminants [4,5]. In the same study, EFSA suggested the need for continued efforts to generate more analytical data on *Alternaria* toxins using sensitive methods to reduce uncertainty in exposure assessments.

**Figure 1:**
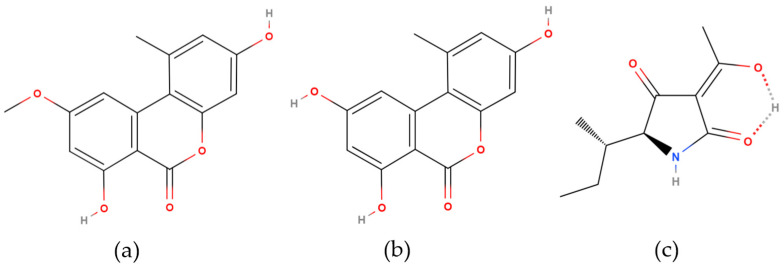
Chemical structures of (**a**) alternariol monomethyl ether (AME), (**b**) alternariol (AOH), and (**c**) the predominant form of tenuazonic acid in organic solvents and water (TeA) according to Mikula et al. (2013) [20]. Structures created with MolView.org.

The toxicity of these mycotoxins extends to acute and chronic adverse effects on microbes, plants, humans, and animals [6,7]. TeA is known for its acute toxicity, evidenced by adverse outcomes in rodents (LD_50_ = 81–186 mg/kg bw) and chicken embryos (LD_50_ = 0.55 mg/egg), as well as in various animal-feeding trials causing vomiting and hemorrhages in lungs and the gastrointestinal system. Furthermore, in vitro studies have demonstrated the cytotoxic effects of TeA, particularly when combined with AME, in human intestinal epithelial cells (Caco-2) and hepatocytes (HepG2) [8]. AOH and AME were demonstrated to be cytotoxic and genotoxic [9]. Moreover, several studies confirmed the ability of AOH and AME to cause DNA damages already at low concentrations [10,11,12,13]. Despite the evident health risks, *Alternaria* toxins remain unregulated in food in any country, though the European Commission has set indicative alert levels for these mycotoxins in specific food commodities, prompting investigations into the presence of *Alternaria* toxins and the impact of food processing on their levels [4,5,14]. Investigation on the metabolism and excretion profiles of *Alternaria* toxins has been limited [15,16,17], with TeA being the only toxin with known toxicokinetic (TK) parameters in pigs and broiler chickens [18] and partially known in humans [19]. The assessment of dietary co-exposure to *Alternaria* toxins is currently based on direct mycotoxin quantification in food. A comprehensive risk assessment at individual level is challenged by the multiple metabolic pathways involved and hampered by the lack of toxicokinetic parameters and health-based evidence values. This challenge is further exacerbated by the diverse chemical groups and physicochemical properties of *Alternaria* toxins, complicating the validation of a unified analytical method, especially considering the peculiar acidity and tautomerism [20] of TeA, as displayed in Figure 1.

Understanding the potential health consequences in humans necessitates the capability of conducting accurate mycotoxin exposure assessments at the individual level and determining population toxicokinetics. In response to this growing need for accurate mycotoxin exposure assessment, human biomonitoring (HBM) has been evolving, with a shift toward methodologies that are less invasive and more patient-friendly. This transition is pivotal for enhancing our capacity to monitor exposure to *Alternaria* mycotoxins in a manner that is both efficient and acceptable to the public. Capillary blood microsampling, particularly using volumetric absorptive microsampling (VAMS), allows home and remote sampling by providing a minimally invasive alternative to the traditional blood withdrawal [21,22]. Microsampling significantly enhances the feasibility of large-scale human biomonitoring (HBM) studies [23] and facilitates more comprehensive pharmaco/toxicokinetic trials [24] by increasing patient participation and enabling more frequent sample collection. Moreover, owing to improvements in the analytical performances in detection and quantification of analytes, the analysis of capillary blood on VAMS, whole blood, serum, and plasma provides comparable results [24,25]. These factors make whole blood microsampling a valid alternative compared to traditional hematic matrices aligning with current trends in analytical methodology that prioritize patient comfort and study feasibility while maintaining analytical rigor. The development of quantitative methods for analyzing multi-mycotoxins in capillary blood, collected via volumetric absorption microsampling (VAMS) techniques such as Mitra^®^ tips, represented a significant advance in this field [25]. As it is known, mycotoxins present a wide range of polarities and diverse physicochemical properties that require compromising the simultaneous quantification of different classes of mycotoxins and/or the sensitivity of the methods. In fact, the quantification method proposed by Vidal et al. (2021) [25] for 24 mycotoxins was not validated for TeA and the sensitivity for AOH and AME is not sufficient for HBM (LOQ_AOH_ = 2.74 ng/mL; LOQ_AME_ = 3.72 ng/mL), especially if considered in relation with the TTC value of the two toxins (2.5 ng/kg bw).

Metabolization of AOH, AME, and TeA were proved to involve phase I and II reactions [15,17,26], indicating the necessity for quantifying not only the parent mycotoxins but also their metabolites for accurate exposure assessment. Aichinger et al. (2020) [27] highlighted the importance of screening for glucuronide metabolites of AOH and AME in urine for effective HBM. This approach implies being able to quantify accurately mycotoxins and their metabolites. Unfortunately, this remains a challenge given the lack of commercially available reference standards for these metabolites. An approach to quantify phase II metabolites is introducing an enzymatic hydrolysis step to convert any conjugate into the parent molecules, as tentatively performed by Asam et al. (2013) [19]. Nonetheless, previous methods have not validated the co-quantification of *Alternaria* toxins, considering the conversion of phase II conjugates [28,29].

HBM is not often performed in human fecal material, although studies in rats demonstrated the scarce bioavailability of AOH and AME that are preferentially excreted through the gastrointestinal tract [30]. Krausová et al. (2021) [31] made advances in detecting multi-mycotoxins in toddler stools, but the method was validated only for AOH and AME, not TeA. In Appendix A, a resume of validation parameters of the mentioned analytical methods for the quantification of one or more *Alternaria* toxin in biological matrices is provided.

In response to these challenges, this study aims to advance HBM of *Alternaria* mycotoxins, offering validated extraction procedures and a state-of-the-art ultra-high performance liquid chromatography coupled with tandem mass spectrometry (UPLC-MS/MS) method. This technique allows for the simultaneous and quantitative biomonitoring of AOH, AME, and TeA, along with their phase II metabolites in urine, capillary blood collected via Mitra^®^ tips (VAMS), and feces. The quantification of phase II conjugates in biological samples is achieved by a double quantification—preparing samples with and without enzymatic hydrolysis employing β-glucuronidase by *Helix pomatia*. This advancement represents a pivotal step toward refining exposure assessments, informing regulatory standards, and ultimately enhancing public health.

## 2. Results and Discussion

### 2.1. Method Optimization

The blood extraction from Mitra^®^ tips (VAMS) was optimized based on the method described by Vidal et al. (2021) [25]. Various extraction solvents were tested, including CH_3_CN, CH_3_OH, CH_3_CN/H_2_O (60/40 *v*/*v*), and CH_3_OH/H_2_O (60/40 *v*/*v*) added with 0, 0.1, and 1% CH_3_COOH. Pure CH_3_OH demonstrated to be the best extraction solvent, as the presence of water resulted in turbid extracts with high chromatographic noise, and the presence of acid negatively affected the recovery of TeA. A novel enzymatic hydrolysis step, based on the method published by Amante et al. (2021) [32], was introduced after the extraction to convert phase II conjugates into the parent molecule. The original sample preparation foresaw the evaporation of the extract and reconstitution of the residue in 50 µL of injection solvent. Following the same procedure, the samples would have an overall four-fold dilution. For this reason, in the optimized procedure, the residues are reconstituted in 25 µL (two-fold dilution) of injection solvent and transferred into glass total recovery vials (Waters, Manchester, UK). Additionally, the centrifugal filtration was replaced with a centrifugation (10 min at 10,000× *g*) to avoid loss of sample since the CH_3_OH extracts resulted cleaner than with the watery extraction solvents.

Urine sample preparation was based on the salting-out assisted liquid–liquid extraction (SALLE) described by Vidal et al. (2018) [33], which was extended to include *Alternaria* toxins without further optimization, as the performances resulted fit for purpose.

**Figure 2:**
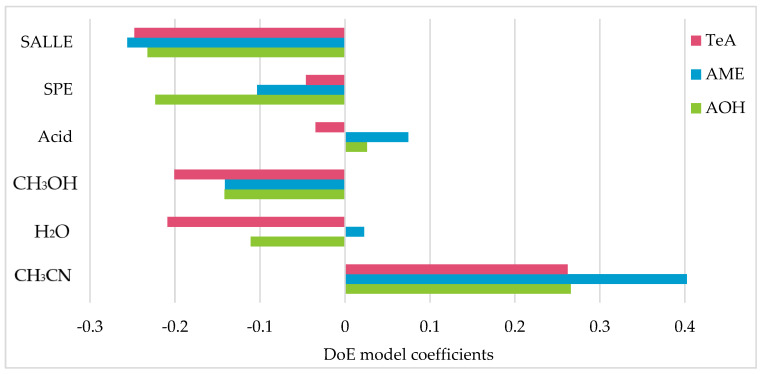
Normalized coefficients for the factors considered during the design of experiments process performed to optimize the extraction of TeA, AME, and AOH from human feces.

The extraction of feces samples was developed using a design of experiments (DoE) approach. The solvents and extractions techniques considered were retrieved from the methods published by Krausová et al. (2021), Lauwers et al. (2019), Miró-Abella et al. (2019), and Puntscher et al. (2019a; 2019b) [16,30,31,34,35]. The DoE, performed using D-Optimal from the Chemometric Agile Tool (CAT) in the R environment [36,37], provided 26 independent experiments with different levels of CH_3_CN, H_2_O, CH_3_OH, CH_3_COOH, use of solid phase extraction (SPE) cartridges (Strata C-18, Phenomenex, Woerden, The Netherlands; Oasis, Waters, Manchester, UK), and use of SALLE. Figure 2 displays the coefficients of the DoE models computed for each analyte using the chromatographic peak area, demonstrating that solid liquid extraction (SLE) with CH_3_CN was the best approach, and the addition of CH_3_COOH positively influenced the extraction of AME and AOH. Accordingly, the selected extraction procedure involved SLE with CH_3_CN containing 1% CH_3_COOH (*v*/*v*).

Finally, a novel liquid chromatography–tandem mass spectrometry (LC-MS/MS) analysis method was developed using an Acquity UPLC system coupled to a Waters Xevo^®^ TQ-XS tandem quadrupole mass spectrometer (Waters, Manchester, UK) equipped with an electrospray interface. MS/MS analysis was carried out using multiple reaction monitoring (MRM) with negative electrospray ionization (ESI^−^). The MRM parameters for ionization and fragmentation were optimized by tuning the compounds of interest. For the three *Alternaria* mycotoxins, the formation of the [M-H]^−^ quasi-molecular ion led to higher precursor ion intensities. The two most intense product ions were selected for the MRM transitions in the MS method after applying different collision energies.

The mobile phase (mp) composition, flow, gradient, column type, injection volume, and strong and weak wash solvent composition and volumes were evaluated based on peak shape, area, and signal-to-noise ratios. Optimal separation and sensitivity was obtained using 1% CH_3_COOH (*v*/*v*) in H_2_O (mp A) and pure CH_3_CN (mp B) and alternatively with H_2_O/CH_3_OH/CH_3_COOH (94/5/1, *v*/*v*/*v*, mp A) and CH_3_OH/H_2_O/CH_3_COOH (97/2/1, *v*/*v*/*v*, mp B) adjusted with 5 mM of CH_3_COONH_4_. The first set of mps was chosen since it is aligns more closely with green chemistry principles [38]. The column choice was governed mostly by the peak shape of TeA that tended to tail. Overall, the Acquity UPLC^®^ HSS T3 (1.8 μm × 2.1 × 100 mm) column paired with a pre-column Acquity UPLC^®^ HSS T3 VanGuard^TM^ (1.8 µm) (Waters, Manchester, UK) provided the best chromatographic separation and narrowest peaks. Moreover, accordingly with the lower volume of blood samples available for the analysis, the loop overfilling factor of the Acquity UPLC system was set to 1.5.

### 2.2. Method Validation

The validation of analytical methods for the quantification of Alternaria toxins—AOH, AME, TeA—and their phase II conjugates in human urine, blood, and feces was conducted with a focus on ensuring sensitivity, recovery, and applicability across the biological matrices considered. The calibration model was fit by applying a quadratic 1/x weighed fitting. The performance of the methods were demonstrated through a range of metrics including the coefficient of determination (R^2^), limit of detection (LOD, ng/mL), calibration range - expressed as the lower limit of quantification (LLOQ, ng/mL)–upper limit of quantification (ULOQ, ng/mL) interval - signal suppression enhancement (SSE, %), apparent recovery (R_A_, %), and extraction efficiency (R_E_, %), recovery (%), repeatability (RSD_r_, %), and intermediate precision (RSD_R_, %). Table 1 summarizes these performance indicators, underscoring the robustness of the method across all tested matrices. The validation results demonstrate robust linearity and goodness-of-fit with R^2^ above the acceptance threshold (>0.990) The LODs obtained in urine and capillary blood were notably <0.1 and <0.05 ng/mL, confirming the feasibility of the calibration range chosen (0.1–10 ng/mL for urine and 0.05–10 ng/mL for blood) and affirming the sensitivity of the method. For fecal samples, higher LOD values (0.204–0.424 ng/mL) were obtained reflecting the complexity of the matrix and associated chromatographic noise, yet remain coherent within the calibration range. The LLOQ for the three analytes was set as the concentration of the lowest calibration point and verified for recovery, repeatability, and intermediate precision. Accordingly, the obtained recovery (%), repeatability (RSD_r_, %), and intermediate precision (RSD_R_, %) were within the acceptable ranges for the three matrices. No interference peaks and carry-over were detected at the retention time ± 2.5% of the analytes with S/N values ≥3. The introduction of the enzymatic hydrolysis, achieved by the use of β-glucuronidase by *Helix pomatia* on the urine and blood extract, did not influence negatively the performances of the method. The methods were successfully validated and obtained results comparable with the procedures without hydrolysis. Remarkably, the LOD values obtained with hydrolysis were slightly lower than the ones without hydrolysis, confirming the suitability of the approach.

### 2.3. Comparison with Previously Published Methods

The validation of our methods presents significant improvements over previous studies, particularly in the detection limits for Alternaria toxins in human biological samples. Comparing the LOD values obtained for AME and AOH in urine with the existing literature [28,29], the results align well, broadcasting similar sensitivities. Notably, the present study marks a significant improvement in detecting TeA, with LODs (0.016 ng/mL in urine; 0.048 ng/mL in urine after hydrolysis) an order of magnitude lower than previously published methods [19,28,30]. This enhancement in sensitivity indicates a substantial advancement in our ability to detect and quantify TeA at low concentration and is crucial for accurately assessing low levels of exposures that are greatly influencing left-censored data. Similar conclusions can be drawn for feces samples; the present method shows comparable or improved performance relative to those validated by Krausová et al. (2021) [31] in infant feces and Puntscher et al. (2019) [30] in rat feces, indicating its applicability in complex matrices and providing a new tool to investigate HBM in this type of matrix. The method optimization for capillary blood, collected via Mitra^®^ tips, extends the sample preparation technique of Vidal et al. (2021) [25] to include TeA and enhances the quantification performance for AOH and AME. Overall, the sensitivity of the methods presented opens the possibility to detect and quantify Alternaria toxins in samples of subjects exposed at or below the TTC. Moreover, the introduction of the hydrolysis step allows for the quantification of both parent Alternaria toxins and their phase II conjugates in urine and capillary blood. In fact, the enzyme chosen provides glucuronidase and sulfatase activity that can be exploited for the comprehensive assessment of total toxin exposure, including glucuronidated and sulfonated forms of Alternaria toxins that are extensively described in the literature [15,17,26,27]. The successful validation of a quantification method for phase II conjugates in various biological matrices is a significant advancement in HBM of Alternaria toxin. By accounting for both parent compounds and their conjugated forms, the developed method provides an accurate tool to assess exposure and to possibly investigate their toxicokinetics, which is essential for conducting reliable risk assessments and establishing regulatory standards.

### 2.4. Matrix Effects

Extraction efficiency was acceptable for all mycotoxins extracted from the three matrices, except for AME in urine prepared without enzymatic hydrolysis (25.7%). The investigation of the signal suppression enhancement showed diminishment of the signals of AME in urine prepared with enzymatic hydrolysis and AOH in urine prepared without enzymatic hydrolysis, 24.5% and 22.1%, respectively. To compensate for the matrix effects, isotope-labeled AME (^13^C_15_-AME) was added as internal standard (IS) to the samples before the extraction. The apparent recovery, achieved by considering the responses, i.e., the ratio of the areas of the analyte and the internal standard corrected by the concentration of the internal standard, was ensured within 92.6–108.9% for the three toxins for all the extraction procedures. This demonstrates that any losses due to incomplete extraction or SSE were proportional systematic errors [39]. Therefore, the addition of ^13^C_15_-AME as an internal standard accounted for the losses, thus preserving the overall recovery.

### 2.5. Stability

Stability tests for urine and capillary blood collected via VAMS Mitra^®^ tips are reported in Table 2. The three toxins did not show any sign of degradation in urine, with recovery ranging 87.0–116.0% considering all the conditions tested. Vidal et al. (2021) [25] reported no loss of AME and AOH at 10 ng/mL after storing of the capillary blood for 7–21 days at 4 °C or at room temperature (recovery ranging between 83.6 and 118.0%). The stability test performed revealed no significant loss of AME, confirming the same results. On the contrary, the recovery obtained for AOH after 5 days of storage at 20 °C was 64.1% and substantially differing from the one reported in the literature [25], namely 118%. TeA is generally stable in both capillary blood and urine; however, the recovery after storage at 20 °C for 5 days for the capillary blood at 10 ng/mL was reduced to 76.5%. Overall, the stability of the three toxins in capillary blood on Mitra^®^ tips and urine is good, since no extensive decline in the recovery was proven. Avoiding the storage of capillary blood samples collected via VAMS Mitra^®^ tips at room temperature or higher, however, is advisable to circumvent any loss of TeA and AOH.

### 2.6. Application to Real Samples

The validated methods were applied to real samples obtained from a small-scale exposure study on three volunteers that consumed a watery bolus containing one of the three Alternaria toxins investigated at the TTC.

TeA was detectable between 15 min and 4 h from the exposure in blood collected via VAMS Mitra^®^ tips (*n* = 5) and between 1 and 8 h in urine (*n* = 6). The maximum concentrations of TeA were 0.40 and 29.37 ng/mL, in blood and urine, respectively. Notably, the hydrolysis step led to an increase in the concentrations of TeA, leading to a maximum concentration of 1.04 ng/mL in blood and 31.00 ng/mL in urine. Due to the lower TTC of AOH and AME, these toxins were quantified at lower concentrations compared to TeA. AOH was detectable after 23 h in six blood samples of which four could be quantified; the maximum concentration found was 0.18 ng/mL before hydrolysis. AOH was detected in three samples in urine between 23 and 39 h from the exposure, but was quantifiable only in one with a concentration of 0.12 ng/mL before hydrolysis. Similar to TeA, an increment in the concentrations followed the application of the hydrolysis; the maximum concentration for total AOH was 0.27 ng/mL in blood and 0.16 ng/mL in urine. Additionally, AOH could be quantified in one more urine sample after hydrolysis (0.11 ng/mL). AME was detected in several blood samples collected (*n* = 7), and quantified in four of them with a maximum concentration of 0.16 ng/mL before hydrolysis and 0.21 ng/mL after hydrolysis. No urine sample contained AME above the LLOQ, only after hydrolysis two samples were quantified at 0.11 and 0.12 ng/mL. Regarding the analysis of stool samples, AME was detected for the first time after 35 h from the exposure at a concentration of 7.86 ng/g of dry matrix. On the contrary, TeA and AOH were not detected. While this was an expected outcome for TeA, since a study in rats demonstrated that only up to 2% of the dose administered is excreted via the gastro-intestinal tract, AOH was supposed to be detected since its excretion in the same study was of 89% of the dose administered in this matrix [30]. However, the only sample provided by the volunteer was collected just 13 h after the ingestion of AOH, which was probably an insufficient time frame between the exposure and the collection to record the excretion.

The concentrations of AOH and AME after exposure at a dose equal to the TTC reached levels at the limit of the validated method. However, the results demonstrated the applicability of the method for direct risk assessment and large-scale HBM. On the other hand, the concentrations obtained for TeA meet perfectly the validation range of the methods, thus opening the possibility for a broader toxicokinetic study. Moreover, the data highlight the crucial role of the hydrolysis step in accurately assessing the content of *Alternaria* toxins in urine and blood, as it allows for the quantification of both free and conjugated forms of the toxins. This represents an important novelty and aligns with the opinion of the scientific community in the context of the HBM of *Alternaria* toxins [27].

## 3. Conclusions

In conclusion, the developed and validated UPLC-MS/MS method represents a significant advancement in the biomonitoring of *Alternaria* toxins in human biological matrices. This multi-matrix approach, which includes urine, capillary blood, and feces, provides a comprehensive tool for assessing exposure to AOH, AME, TeA, and their phase II conjugates. The incorporation and validation of an enzymatic hydrolysis step using β-glucuronidase is a novel aspect that allows a more accurate quantification of toxin levels by converting phase II conjugates back to their parent compounds, ensuring a more realistic representation of exposure and avoiding underestimation [27].

The validated methods showed improved sensitivity for AOH and AME, and included for the first time the quantification of TeA in capillary blood and human feces, addressing critical gaps in current research. The methods were successfully applied to real samples collected during a small-scale toxicokinetic study, confirming their applicability for risk assessment. Moreover, the results showed the potential for determination of the toxicokinetic properties of TeA through carefully designed human toxicokinetic trials, further enhancing our understanding of its behavior in the human body. The lower LODs achieved are crucial for generating reliable exposure data to inform regulatory standards and public health authorities. By providing left-censored data at an acceptable level and operating on multiple matrices, the method enables the possibility to perform accurate HBM and comprehensive risk assessment associated with *Alternaria* toxin exposure. Additionally, the application of minimally invasive microsampling techniques, such as the use of Mitra^®^ tips for capillary blood collection, aligns with the trend toward less invasive HBM methods and facilitates the implementation of large-scale studies.

In summary, the development of a multi-matrix UPLC-MS/MS method, coupled with the application of minimally invasive microsampling techniques, represents a significant step forward in the HBM of *Alternaria* toxins. It provides the precise quantification of these natural toxins in different matrices, promoting the possibility of conducting comprehensive risk assessment and large-scale HBM studies, ultimately contributing to the protection of public health.

## 4. Materials and Methods

### 4.1. Chemicals and Reagents

One milligram of the individual mycotoxin (AOH, AME, and TeA, Figure 1) solid standards was obtained from Fermentek (Jerusalem, Israel), while 25.1 µg of isotope-labeled AME (^13^C_15_-AME) as internal standard (IS) was purchased from Merck (Darmstadt, Germany). All the mycotoxins and isotope-labeled mycotoxins solid standards were dissolved in methanol (CH_3_OH) to reach the final concentration of 1 mg/mL and 25.1 µg/mL, respectively, and were stored at −20 °C. From the individual stock solutions, a standard mixture of TeA, AME, and AOH, 10 ng/mL each, and a ^13^C_15_-AME solution of 50 ng/mL were prepared in CH_3_OH. Water (H_2_O) was obtained from an Aurim^®^ Pro water system from Sartorius (Brussels, Belgium). LC-MS grade (99.95%) methanol (CH_3_OH) and acetonitrile (CH_3_CN) were purchased from BioSolve (Valkenswaard, The Netherlands). Glacial acetic acid (CH_3_COOH, 100%), formic acid (HCOOH, 98–100%), ammonium acetate (CH_3_COONH_4_, 99%), and sodium acetate (CH_3_COONa, 99%) were supplied by Merck (Darmstadt, Germany). Sodium chloride (NaCl, 99%) and magnesium sulfate (MgSO_4_, 99%) were obtained from VWR Chemicals (Leuven, Belgium). β-glucuronidase by *Helix pomatia* type HP-2 aqueous solution (≥100.000 units/mL) was purchased from Merck (Darmstadt, Germany). VAMS Mitra^®^ tips were obtained from Trajan Scientific and Medical (Torrance, CA, USA).

### 4.2. Sample Collection

EDTA-anticoagulated blood samples for method development and validation purposes were supplied by Rode Kruis Vlaanderen (Ghent, Belgium). The blood samples were aliquoted in 0.5 mL cryogenic tubes and stored at −80 °C. Once the blood was defrosted and homogenized, the samples were prepared by dipping the tip into the whole blood. After completely filling the tips, the devices were dried in the accompanying clamshells for one night at room temperature and stored at −80 °C until the day of the sample preparation. Individual urine samples were donated by 20 healthy subjects in plastic containers and pooled together. The samples were stored at −20 °C until the day of the sample preparation. Individual feces samples were donated by 8 healthy subjects in plastic containers equipped with screw caps with spoon and pooled together. The samples were freeze-dried for 24–36 h at 0.300 mbar (Büchi, Uster, Switzerland) and stored at −80 °C until the day of the sample preparation. Real samples were obtained via a small-scale human exposure study performed as described by Vidal et al. (2018) and Visintin et al. (2023) [17,33]. Briefly, three volunteers ingested a watery bolus containing either TeA or AOH, or AME at the TTC, after a washout period of 2 days. The volunteers were then asked to collect individual blood (via VAMS Mitra^®^ tips), urine, and fecal samples for 48 h. The blood was collected following a schedule: at 15, 30 min, 1, 2, 4, 6, 8, 12, 24, 36, and 48 h after the bolus consumption. Throughout the study, the volunteers followed a nutritional regime developed specifically for minimizing the dietary intake of *Alternaria* toxins. Samples were aliquoted and stored at −80 °C until the day of the sample preparation. The study was conducted according to the guidelines in the Declaration of Helsinki and was approved by the Ethical Committee of the Ghent University Hospital (B670201630414). Informed consent was obtained from all the volunteers before participation.

### 4.3. Sample Extraction

#### 4.3.1. Capillary Blood Samples

The blood extraction from Mitra^®^ tips (VAMS) was performed as described by Vidal et al. (2021) [25] after optimization of the extraction solvent. After separation of the polymeric tips from the plastic handlers, the tips were transferred into Eppendorf Safe-Lock Tubes 1.5 mL (Eppendorf Belgium N.V.-S.A., Aarschot, Belgium). The calibration curves were built by spiking 9 tips at different concentration levels with the mycotoxin standard mix and IS solutions. The final concentrations were 0.00 (blank), 0.05, 0.10, 0.20, 0.50, 1.00, 2.00, 5.00, and 10.00 ng/mL for AOH, AME, and TeA, and 2.50 ng/mL for the IS. The tips were left air-drying in a cool and dark place to ensure the complete absorption of the mycotoxins on the polymeric material. The extraction was carried out by adding 550 µL of CH_3_OH, ultrasonicating for 20 min at room temperature (Branson Ultrasonic Corporation, Danbury, CT, USA), and shaking the samples for 30 min at room temperature using an overhead shaker (Agilitec, Paris, France). Different extraction solvents (mix of H_2_O and CH_3_CN or CH_3_OH with a different percentage of CH_3_COOH) were considered. Based on matrix effect and recovery, CH_3_OH was eligible as the best extraction solvent for the three mycotoxins. The tips were removed, and the supernatant was split in two aliquots and evaporated under a gentle stream of N_2_ at room temperature using a Turbovap LV Evaporator (Biotage, Charlotte, NC, USA). The extract of the first aliquot was reconstituted in 25 µL of injection solvent (CH_3_CN/H_2_O/CH_3_COOH, 25/74/1, *v*/*v*/*v*), vortexed, and centrifuged for 10 min at 10,000× *g*. Finally, samples were transferred into glass total recovery vials (Waters, Manchester, UK). The extract of the second aliquot was subjected to hydrolysis using β-glucuronidase by *Helix pomatia* to hydrolyze phase II conjugates into the original mycotoxin. The extracts were reconstituted in 250 µL H_2_O and 25 µL acetate buffer 0.05 M pH 5; 5 µL of β-glucuronidase solution was added to the solution, and the tubes were vortexed for 1 min and incubated at 55 °C while shaking at 1400 rpm using a Thermo-Shaker TS-100 (Biosan, Riga, Latvia). The reaction was stopped after 1 h by protein precipitation obtained by adding 250 µL of ice-cold CH_3_CN. The suspension was vortexed for 30 s and centrifuged for 5 min at 10,000× *g*. The supernatant was transferred in a tube and evaporated under a gentle stream of N_2_ at room temperature. The residue was reconstituted in 25 µL of injection solvent (CH_3_CN/H_2_O/CH_3_COOH, 25/74/1, *v*/*v*/*v*), vortexed, and centrifuged for 10 min at 10,000× *g*. Finally, samples were transferred into glass total recovery vials (Waters, Manchester, UK).

#### 4.3.2. Urine

*The urine sample preparation was based on the salting-out assisted liquid–liquid extraction (SALLE) as described by* Vidal et al. (2018) [33]. Briefly, 2 mL of the thawed urine sample was transferred to a 50 mL extraction tube. The calibration curves were built by spiking 9 samples at different concentration levels with the mycotoxin standard mix and IS solutions. The final concentrations were 0.00 (blank), 0.10, 0.20, 0.50, 1.00, 2.00, 5.00, and 10.00 ng/mL for AOH, AME, and TeA, and 1.00 ng/mL for the IS. To the samples, 18 mL of CH_3_CN/H_2_O/HCOOH (52/45/3, *v*/*v*), 4 g of anhydrous magnesium sulfate, and 1 g of sodium chloride were added. The samples underwent 1 min of vortex shaking, 30 min of overhead shaking at room temperature (Agilitec, Paris, France), and centrifugation at 4000× *g* for 10 min (Sigma 3-16PK, Osterode am Harz, Germany). The organic fraction was transferred to a glass tube and evaporated under a gentle stream of N_2_ at 40 °C. The residue was re-dissolved in 250 µL of injection solvent (CH_3_CN/H_2_O/CH_3_COOH, 25/74/1, *v*/*v*/*v*), vortexed, and centrifuged for 10 min at 10,000× *g* (Sigma 3-16PK, Osterode am Harz, Germany). Finally, samples were transferred into UPLC vials.

The enzymatic hydrolysis of the urine samples was performed analogously to the hydrolysis performed on the blood samples. Briefly, 125 µL of the thawed urine sample was transferred into Eppendorf Safe-Lock Tubes 1.5 mL. The calibration curves were built by spiking 8 samples at different concentration levels with the mycotoxin standard mix and IS solutions. The final concentrations were 0.00 (blank), 0.10, 0.20, 0.50, 1.00, 2.00, 5.00, and 10.00 ng/mL for AOH, AME, and TeA, and 1.00 ng/mL for the IS. The samples were combined with 12.5 µL acetate buffer (0.05 M, pH 5) and 2.5 µL of β-glucuronidase, and vortexed for 1 min. After incubation for 1 h at 55 °C while shaking at 1400 rpm using a Thermo-Shaker TS-100 (Biosan, Riga, Latvia), the reaction was stopped by protein precipitation obtained by adding 125 µL of ice-cold CH_3_CN. The suspension was vortexed for 30 s and centrifuged for 5 min at 10,000× *g* (Sigma 3-16PK, Osterode am Harz, Germany). The supernatant was transferred in a tube and evaporated under a gentle stream of N_2_ at room temperature. The residue was reconstituted in 50 µL of injection solvent (CH_3_CN/H_2_O/CH_3_COOH, 25/74/1, *v*/*v*/*v*), vortexed, and centrifuged for 10 min at 10,000× *g* (Sigma 3-16PK, Osterode am Harz, Germany). Finally, samples were transferred into UPLC vials.

#### 4.3.3. Feces

Accordingly to the optimized extraction of Alternaria toxins from feces obtained applying the DoE approach, one gram of freeze-dry feces samples were transferred in 50 mL extraction tubes. Eight blank samples were used as calibrants and spiked at different concentration levels with the mycotoxin standard mix and IS solutions. The final concentrations were 0.00 (blank), 0.63, 1.25, 2.50, 5.00, 7.50, 10.00, and 20.00 for TeA, and 5.00 ng/g for the IS. After 30 min of equilibration in a cool and dark place, the samples were extracted with 20 mL of CH_3_CN/CH_3_COOH (99/1, *v*/*v*). The tubes were vortexed for 1 min, ultrasonicated for 15 min at room temperature (Branson Ultrasonic Corp., Danbury, CT, USA), and overhead shaking at room temperature (Agilitec, Paris, France). After centrifugation for 15 min at 4000× *g*, the supernatant was transferred to a new tube and evaporated under a gentle N_2_ flow. The residues were re-suspended in 250 µL of injection solvent (CH_3_CN/H_2_O/CH_3_COOH, 25/74/1, *v*/*v*/*v*) and vigorously vortexed. Finally, the samples were filtered with Durapore^®^ PVDF 0.22µm centrifugal filters (Merk Millipore, Cork, Ireland) and transferred into UPLC vials.

### 4.4. UPLC-MS/MS Analysis and Method Validation

The optimized UPLC-MS/MS analysis was carried out using a Acquity UPLC system coupled to a Waters Xevo^®^ TQ-XS tandem quadrupole mass spectrometer (Waters, Manchester, UK) equipped with an electrospray (ESI) interface. The detection was performed using multiple reaction monitoring (MRM) with ESI^−^. Chromatographic separation was achieved using an Acquity UPLC^®^ HSS T3 (1.8 μm × 2.1 × 100 mm) column paired with a pre-column Acquity UPLC^®^ HSS T3 VanGuard^TM^ (1.8 µm) (Waters, Manchester, UK). Column and autosampler temperature were set at 35 and 10 °C, respectively. Gradient elution was established with a mobile phase consisting of 1% CH_3_COOH (*v*/*v*) in H_2_O (mobile phase A) and pure CH_3_CN (mobile phase B) at a flow rate of 0.4 mL/min. A run started with an increase in B from 25% to 85% during 3 min with a concave profile, followed by an instant increase to 100% B that was maintained until 4.5 min, after which % B was linearly decreased until 25% at 6 min, finally the column was allowed to re-equilibrate for 1.5 min, resulting in a total run time of 7 min. The optimized MRM parameters and the retention times are reported in Table 3 for each compound. The capillary voltage was set at 0.80 kV; desolvation gas temperature and flow were 600 °C and 1000 L/h. The cone curtain and nebulizer gas flow were 150 L/h and 7.0 Bar, respectively. The nebulizer and curtain gas used was N_2_, while the collision gas was Ar. Masslynx^®^. Targetlynx^®^ software 4.2 (Waters Corp., Milford, CT, USA) was used for data acquisition and processing.

The methods were validated to meet the criteria of European Commission (EC) decision no. 2002/657, Commission Implementing Regulation (EU) no. 2023/2782, and ISO 5725-1:2023 as guidance [40,41,42]. The goodness-of-fit of the linear regression (coefficient of determination, R^2^), limit of detection (LOD), calibration range, recovery (%), intra-day precision (repeatability, RSD_r_, CV %), and inter-day precision (intermediate precision, RSD_R_, CV %) were calculated based on the results of 3 days of analysis in which 3 calibration curves were run each day for each matrix, for a total of 9 samples for each concentration level considered. The linearity and the goodness-of-fit of the linear regression were assessed by calculating the coefficient of determination (R^2^) [43] between the spiked concentrations of the analytes and the corresponding instrument response, which was calculated as the ratio of the analyte peak area to the IS peak area, multiplied by the concentration of the IS. Matrix effects, namely signal suppression enhancement (SSE), apparent recovery (R_A_), and extraction efficiency (R_E_), were assessed following the approach described by Sulyok et al. (2006) [39]. Specificity was determined by evaluating the presence of interfering chromatographic peaks in representative blank samples. The carry-over was evaluated by injecting injection solvent after the highest calibration point into the LC-MS/MS system. The stability was investigated at two concentration levels in duplicates to simulate a high- and low-exposure scenario in urine and capillary blood collected via VAMS Mitra^®^ tips. The urine was spiked and stored at 4 °C for 5 days and −20 °C for 21 days, while the VAMS devices were stored at 20 °C for 5 days and 4 °C for 21 days. The hypothesis of influence of hematocrit (Hct) was previously tested and disproved, meaning that the recovery from VAMS Mitra^®^ tips is not hematocrit [25].

## Figures and Tables

**Table 1:** Results of the method validation for the quantification of alternariol (AOH), alternariol monomethylether (AME) and tenuazonic acid (TeA) in urine, capillary blood collected via VAMS Mitra^®^ tips, and feces. The enzymatic hydrolysis was performed by the use of β-glucuronidase by *Helix pomatia*. The performance of the method is reported as the determination coefficient (R^2^), limit of detection (LOD, ng/mL), calibration range—expressed as the lower limit of quantification (LLOQ, ng/mL)–upper limit of quantification (ULOQ, ng/mL) interval—signal suppression enhancement (SSE, %), apparent recovery (R_A_, %), and extraction efficiency (R_E_, %), recovery (%), repeatability (RSD_r_, %), and intermediate precision (RSD_R_, %). The results were obtained by testing the same calibration curve three times for 3 days (*n* = 9).

Analyte	Matrix	Enzymatic Hydrolysis	R^2^	LOD	Calibration Range	SSE	R_A_		R_E_	Recovery		RSD_r_		RSD_R_	
					LLOQ–ULOQ		without IS	with IS		LLOQ	ULOQ	LLOQ	ULOQ	LLOQ	ULOQ
				ng/mL *	ng/mL *	%	%	%	%	%	%	%	%	%	%
AOH	Urine	No	0.9960	0.053	0.1–10	22.1	15.5	94.71	70.3	107.2	100.9	14.0	2.0	0.7	0.6
AME	Urine	No	0.9989	0.050	0.1–10	73.0	18.8	96.3	25.7	105.5	100.3	10.0	5.0	7.4	3.1
TeA	Urine	No	0.9971	0.016	0.1–10	104.1	95.4	95.8	95.7	107.6	98.3	4.0	4.1	3.2	4.7
AOH	Urine	Yes	0.9983	0.042	0.1–10	101.3	64.4	93.55	63.6	102.2	103.1	19.0	3.5	3.0	1.9
AME	Urine	Yes	0.9994	0.043	0.1–10	24.5	24.8	108.9	101.4	103.6	100.9	16.5	4.3	15.4	4.5
TeA	Urine	Yes	0.9983	0.048	0.1–10	80.3	80.01	97.12	99.6	96.22	101.1	11.5	2.1	17.1	1.9
AOH	Blood	No	0.9988	0.027	0.05–10	93.6	89.6	92.6	95.7	106.7	100.1	10.0	2.0	7.7	0.8
AME	Blood	No	0.9967	0.007	0.05–10	62.9	63.3	93.3	100.4	111.1	99.6	11.1	2.3	2.4	1.5
TeA	Blood	No	0.9984	0.029	0.05–10	93.6	89.6	105.6	95.7	108.9	99.6	8.9	1.5	8.8	1.5
AOH	Blood	Yes	0.9963	0.01	0.05–10	70.2	43.3	97.8	61.6	96.3	101.1	6.3	0.4	4.5	4.4
AME	Blood	Yes	0.9957	0.003	0.05–10	113.8	88.8	102.6	78.0	103	102.4	17.8	0.6	12.3	2.1
TeA	Blood	Yes	0.9912	0.026	0.05–10	105.3	64.9	99.6	61.6	98.0	101.1	19.6	3.1	9.5	3.1
AOH	Feces	No	0.9934	0.412	1.25–20	91.7	77.2	98.4	78.9	103.9	100.3	1.2	0.1	4.2	3.5
AME	Feces	No	0.9919	0.424	1.25–20	82.0	74.9	99.2	89.7	94.5	94.4	1.3	5.0	8.3	5.3
TeA	Feces	No	0.9946	0.204	0.63–20	69.3	62.3	100.1	75.2	101.1	102.1	5.9	3.7	5.9	3.7

* for feces, the concentration is expressed as ng/g of dry sample.

**Table 2:** Results of the stability test of alternariol (AOH), alternariol monomethylether (AME), and tenuazonic acid (TeA) in urine and capillary blood collected via VAMS Mitra^®^ tips.

Analyte	Stability in Urine (%)				Stability in Capillary Blood (%)			
	0.1 ng/mL	10 ng/mL	0.1 ng/mL	10 ng/mL	0.05 ng/mL	5 ng/mL	0.05 ng/mL	5 ng/mL
	−20 °C 21 Days	−20 °C 21 Days	4 °C 5 Days	4 °C 5 Days	4 °C 21 Days	4 °C 21 Days	20 °C 5 Days	20 °C 5 Days
AOH	93.0	99.5	87.0	89.8	104.5	94.9	99.2	64.1
AME	115.0	95.8	107.0	95.9	108.9	111.3	104.0	93.9
TeA	101.6	101.0	116.0	96.2	101.2	99.9	89.6	76.5

**Table 3:** Multiple reaction monitoring (MRM) parameters used for the quantification of alternariol (AOH), alternariol monomethylether (AME), and tenuazonic acid (TeA). Retention time (Rt), cone voltage, mass-to-charge ratio of quasi-molecular ion ([M-H]^−^ *m*/*z*), mass-to-charge ratio of the product ions m/z, and collision energy (CE).

Analyte	Rt	Cone	[M-H]^−^ *m*/*z*	CE	Product Ion *m*/*z*
	(min)	(V)		(eV)	
AOH	2.80	73	257.0	23	213.0
				25	215.0
TeA	2.68	50	196.1	23	112.1
				18	139.0
AME	3.44	60	271.1	31	255.0
				22	256.0
^13^C_15_-AME	3.44	60	286.1	31	269.0
				22	270.0

## Data Availability

The raw data supporting the conclusions of this article are available from the authors on request.

## Appendix A

**Table A1:** Resume of validation parameters from published analytical methods for the quantification of one or more *Alternaria* toxin in biological matrices. The parameters reported are limit of detection (LOD, ng/mL or ng/g), limit of quantification (LOQ, ng/mL or ng/g), calibration range (ng/mL or ng/g), extraction efficiency (R_E_, %), intermediate precision (RSD_R_, %), repeatability (RSD_r_, %), signal suppression enhancement (SSE, %), signal suppression enhancement with internal standard compensation (SSE_IS_, %), and apparent recovery (R_A_, %).

Reference	Matrix	Analyte	LOD	LOQ	Linear Range	R_E_	RSD_R_	RSD_r_	SSE	SSE_IS_	R_A_
			ng/mL	ng/mL	ng/mL	%	%	%	%	%	%
De Ruyck et al. (2020) [28]	Human urine	AME	0.037	0.063							
		AOH	0.015	0.061							
		TeA	0.180	0.250							
Asam et al. (2013) [19]	Human urine	TA-DNPH	0.2	0.6	1–100		6.7				
Krausová et al. (2021) [31]	Infant feces *	AOH	0.8	1.6	10–30	68–78	28–8	40–9	96		
		AME	0.03	0.06	0.7–2.2	40–49	27–16	23–7	83		
Vidal et al. (2021) [25]	Humanblood (VAMS)	AOH	1.37	2.74	10–250				13.2	105	136–92.8
		AME	1.86	3.72	10–100				10.4	83.1	124–118
Martins et al. (2019) [29]	Human urine	AOH	0.4	0.9	0.1–50		7.8	5.9			89.7
		AME	0.5	1	0.1–50		9.6	8.2			87
Puntscher et al. (2019) [30]	Rat urine	AOH	0.05	0.1	0.1–100	43	8	8	62		
		AME	0.005	0.01	0.02–20	31	9	8	66		
		TeA	0.1	1	1.2–1200	117	17	7	86		
	Rat feces **	AOH	0.5	1	1–1000	65	19	11	42		
		AME	0.3	0.6	0.2–200	56	18	7	19		
		TeA	30	60	12–12,000	89	23	13	109		

* LOD, LOQ, and linear range are expressed as ng/g. ** LOD, LOQ, and linear range are expressed as ng/g of dry feces after conversion from ng/mL of dry blank feces suspended in water (1:10), which accounted for a ten-fold higher spiking concentration.
